# Evaluation of matrix-assisted laser desorption ionization-time of flight mass spectrometry (MALDI-TOF MS) for identification and clustering of *Neisseria gonorrhoeae*

**DOI:** 10.1186/s12866-015-0480-y

**Published:** 2015-07-24

**Authors:** Anna Carannante, Elena De Carolis, Paola Vacca, Antonietta Vella, Caterina Vocale, Maria Antonia De Francesco, Marco Cusini, Simonetta Del Re, Ivano Dal Conte, Antonio Cristaudo, Patrizia Ober, Maurizio Sanguinetti, Paola Stefanelli

**Affiliations:** Departmentof Infectious, Parasitic & Immune-mediated Diseases, Istituto Superiore di Sanità, Rome, Italy; Istituto di Microbiologia, Università Cattolica del Sacro Cuore, Rome, Italy; Unit of Clinical Microbiology, CRREM Laboratory, St. Orsola-Malpighi, University Hospital, Bologna, Italy; Dipartimento di Medicina Molecolare e Traslazionale, Sezione di Microbiologia, University of Brescia, Brescia, Italy; Fondazione IRCCS Ca’ Granda Ospedale Maggiore Policlinico, Milan, Italy; Microbiology and Virology Laboratory, Infectious Diseases, Amedeo di Savoia Hospital, Turin, Italy; MD, Dip-GUM. STI Clinic, Department of Infectious Diseases, Amedeo di Savoia Hospital, Turin, Italy; San Gallicano Dermatologic Institute-IRCSS-Rome, Rome, Italy; Microbiology and Virology Laboratory, Santa Chiara Hospital, Trento, Italy

**Keywords:** *Neisseria gonorrhoeae*, MALDI-TOF MS, G1407, Cluster

## Abstract

**Background:**

The sexually transmitted infection gonorrhea remains a public health concern for becoming resistant to drug treatments available. The purpose of this study was to evaluate the usefulness of the matrix-assisted laser desorption ionization-time of flight mass spectrometry (MALDI-TOF MS) to identify and cluster *Neisseria gonorrhoeae*.

From a current monitoring in Italy, as part of the European Gonococcal Antimicrobial Surveillance Programme (EURO-GASP), 93 gonococci collected from 2007 to 2012 susceptible (44 isolates) and resistant (49 isolates) to cefixime were selected. Minimum Inhibitory Concentration (MIC) values for cefixime was assessed by Etest carried out in agreement with the manufacturer’s instructions and interpreted referring to European Committee on Antimicrobial Susceptibility testing (EUCAST) clinical breakpoints criteria. Data obtained by *N. gonorrhoeae* multiantigen sequence typing (NG-MAST) and the dendrogram based on the concatenation of *porB* and *tbpB* genes were evaluated. MALDI-TOF MS, to reconfirm gonorrhea identification, analyzed single colonies from freshly grown isolates and applied directly on a ground-steel MALDI target plate. For the MALDI-TOF dendrogram cluster analysis, MSPs (Main Spectrum Profile) from each isolate were created acquiring 5000 shots from 10 technical replicates obtained from bacteria extraction.

**Results:**

Molecular typing by NG-MAST showed 28 sequence types (STs); G1407 was the predominant accounting for 75 gonococci. All the 93 gonococci, except one, were correctly identified at species level by MALDI-TOF MS and G1407 isolates were divided into two clusters.

**Conclusion:**

MALDI-TOF MS for a real-time detection and cluster analysis of gonorrhea is a promising tool for surveillance purposes. Moreover, additional studies are required to collect more data on the performance of MALDI-TOF MS for gonococci.

## Background

*Neisseria gonorrhoeae* is responsible for causing gonorrhea, one of the most common bacterial sexually transmitted disease worldwide, affecting 106 million people each year [1]. Although significant progress was made in reducing the incidence of the disease, gonorrhea infection is becoming a much more difficult to treat, increasing morbidity and medical cost [2–4]. In recent years, the emergence of multi-drug resistant (MDR) *N. gonorrhoeae* is a serious problem for treatment options [3, 5–7]. In particular, as reported by the European Centre for Disease Prevention and Control (ECDC), the proportion of gonococci with reduced susceptibility to cefixime, was 5.1 % in 2009 and 7.6 % in 2011, respectively [3, 8]. Sporadic cases due to ceftriaxone-resistant isolates have also been described in Europe [9, 10].

Surveillance of antimicrobial resistance (AMR) and MDR gonorrhea, promoting susceptibility testing and studying the genetic mechanism of resistance, is a key point for the control strategy and to combat the spread of resistant gonococci [10–14].

In this regard, the rapid diagnosis and typing should be pursued to enhance surveillance capabilities [15–17].

Currently, one of the reference method for gonorrhea typing is the *N. gonorrhoeae* multiantigen sequence typing (NG-MAST). Many reports highlight the NG-MAST highly discriminatory power, reproducibility, speed, low cost and easy to perform; a public database [http://www.ng-mast.net] for analysis and assignment of the allele numbers and Sequence Type (ST) is available, [15, 17]. Nowadays, the ST1407, the main ST together with all the related STs, defined the Genogroup (G) 1407 [14–16]. Gonococci belonging to ST1407, mainly associated with reduced susceptibility and resistance to third generation cephalosporins, are spreading in Europe [13–16] and worldwide [18]. In 2011, it was noted in 20/21 European countries and the association among ciprofloxacin resistance, decreased susceptibility to cefixime and G1407 has been observed [15]. Moreover, isolates belonging to G1407 with an increased minimum inhibitory concentrations (MICs) to ceftriaxone and azithromycin, have also been reported [15]. Overall, the identification of a specific genogroup may predict patterns of antimicrobial resistant among circulating gonococci [16]. Recently, the identification by the matrix-assisted laser desorption ionization-time of flight mass spectrometry (MALDI-TOF MS) has revolutionized the workflow of microbiology laboratories [19, 20], because of its capability to quickly identify bacterial species and yeasts accurately in comparison with conventional methods [21, 22]. Nowadays, MALDI-TOF MS is considered a reliable tool for the detection and typing of bacteria [23–25].

The purpose of this study was to evaluate the usefulness of MALDI-TOF MS to identify and cluster *Neisseria gonorrhoeae*.

## Methods

### *N.gonorrhoea isolates*, microbiological methods and patients

Primary isolation, identification and collection, following standard microbiological procedures of 93 gonococci, collected from 2007 to 2012, were performed by the Collaborating Laboratories belonging to Universities, Sexual Transmitted Infectious (STI) and Dermatology-Venereology (DV) Clinics. Routine diagnostic testing consisted with culture of *N. gonorrhoeae* from clinical samples (*i.e.* urethral exudate) inoculating directly on Thayer-Martin medium (Oxoid Ltd, Milan, Italy) with 1 % IsoVitalex (Oxoid, Hampshire, United Kingdom) at 37 °C in a 5 % CO_2_ atmosphere. Identification was carried out mainly by using API NH system gallery (bioMérieux, Hantverksvägen, Sweden). Isolates were sent to the Istituto Superiore di Sanità (ISS) for storage - 80 °C and further microbiological investigations.

Antimicrobial susceptibility tests were performed following the European Gonococcal Antimicrobial Surveillance Programme (EURO-GASP), [26]. In particular, antimicrobial susceptibility to cefixime, was assessed by Etest (bioMérieux, Hantverksvägen, Sweden) in agreement with the manufacturer’s instructions. MIC values were interpreted referring to European Committee on Antimicrobial Susceptibility testing (EUCAST) clinical breakpoint criteria (S ≤ 0.125 mg/L; R > 0.125 mg/L), (version 5.0, 2015), [27]. The World Health Organization (WHO) *N. gonorrhoeae* G, K, M, O, and P control strains were used in each test [28]. The 93 gonococci were selected referring to antimicrobial susceptibiltiy to cefixime; in particular, 44 isolates were susceptible and 49 resistants. No patient identification was available at the ISS. The isolates were collected for surveillance purposes, thus this work was considered for public health practice, as routine in clinical setting, and no ethical approval was required.

### NG-MAST analysis

Chromosomal DNA was extracted using the QIAamp DNA minikit (Qiagen, Hilden, Germany) according to the manufacturer’s instructions. For NG-MAST analysis, sequence of *porB* and *tbpB* genes were performed using primers and amplification parameters, as previously described [29]. The *porB* and *tbpB* alleles were assigned at the NG-MAST website (http://www.ng-mast.net), following the interpretative criteria [29]. Closely related STs were clustered using published definitions [15, 16]. In particular, all STs which shared one allele and showed >99 % similarity in the other allele (≤5 bp difference for *porB* and ≤4 bp for *tbpB*), were included in the same genogroup [15].

NG-MAST data were analyzed by Unweighted Pair Group Mean Average (UPGMA) clustering method using MEGA software (version 5.2, http://www.megasoftware.net); the distance matrices were based on a bootstrap test of 500 replicates; the evolutionary distances were computed using the Tajima-Nei method and visualized as a dendrogram.

### MALDI-TOF MS and data analysis

For MALDI-TOF MS identification, single colonies from freshly grown isolates were picked and directly applied in duplicate on a ground-steel MALDI target plate. One microliter of a saturated solution of α-cyano-4-hydroxy cinnamic acid, HCCA (Bruker Daltonik, Bremen, Germany) matrix was deposited on the sample tested and allowed to co-crystallize at room temperature.

Spectra useful for identification were recorded in positive linear mode by a Microflex LT mass spectrometer with a laser frequency of 20 Hz; ion source 1 and 2 voltage, 20 kV and 16.7 kV respectively; lens voltage, 8.5 kV After the automatic acquisition spectra were identified by MALDI Biotyper RTC software, database version 3.1 (4613 entries). For instrument calibration bacterial test standard BTS255343 from Bruker Daltonics it has been used. For the evaluation of the identification results we adopted criteria suggested by the manufacturer; briefly, score values below 1.7 indicated a not reliable identification, between 1.7 and 1.99 a probable genus identification and equal or above 2.0 a secure genus identification and probable or highly probable species identification (between 2.0 - 2.29 and above 2.3, respectively). For the MALDI-TOF dendrogram, 93 MSP (Main Spectrum Profile) of the *N. gonorrhoeae* isolates were created to acquire at least 20 separate spectra obtained from 240 shots for a total of above 5000 shots from 10 technical replicates from bacteria extraction with formic acid and acetonitrile, as reported by manufacturer [30]. Quality control peaks was performed using flexAnalysis software (Version 3.3); spectra with outlier peaks or anomalies were removed from the set of the isolate; peaks from 3000 to 10,000 Da were selected and checked so that the allowed difference between the smallest and the largest mass was always lower than 500 ppm. Software settings for MSP creation were those suggested by the Bruker Biotyper 3.1 Version [31]. Reproducible spectra profiles was assessed by composite correlation index analysis (CCI) as a representative value of around high conformance of the spectra [32] The hierarchical cluster analysis was performed with the integrated statistical tool Matlab 7.1 of the BioTyper 3.0 software package using default correlation function. Basing on the values obtained from the pairwise comparison of different spectra, a dendrogram was generated allowing the visualization of similarities among spectra profiles.

## Results

### NG-MAST analysis

**Fig. 1:**
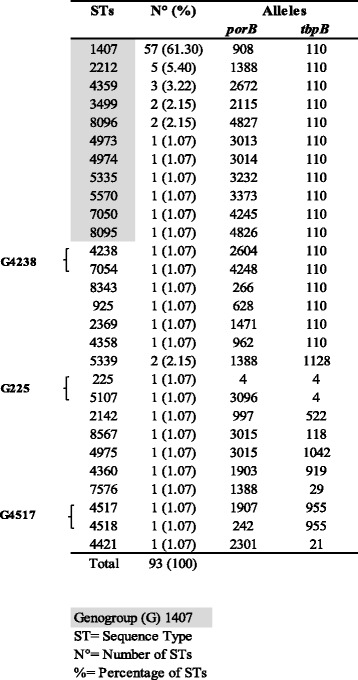
*Neisseria gonorrhoeae* multiantigen sequence typing (NG-MAST) of 93 gonococci isolated from 2007 to 2012 in Italy

The molecular typing identified 28 STs out of the 93 isolates. Figure 1 shows the STs found among G1407. In particular, ST1407 was found in 57 isolates (61.3 %), with *porB908* and *tbpB110* alleles. According to the genogroup definition, and basing on sequence similarity of the *porB* and *tbpB* alleles, ST 2212, 4359, 3499, 8096, 4973, 5570, 7050, 4974, 5335 and 8095 were included in G1407 (75 isolates). The remaining 18, showed different STs, as singletons, except for two belonging to the same ST5339. Out of 18 isolates, 6 belonged to 3 different genogroups, according to the definition [15]: G225, G4238, G4517 (Fig. 1).

**Fig. 2:**
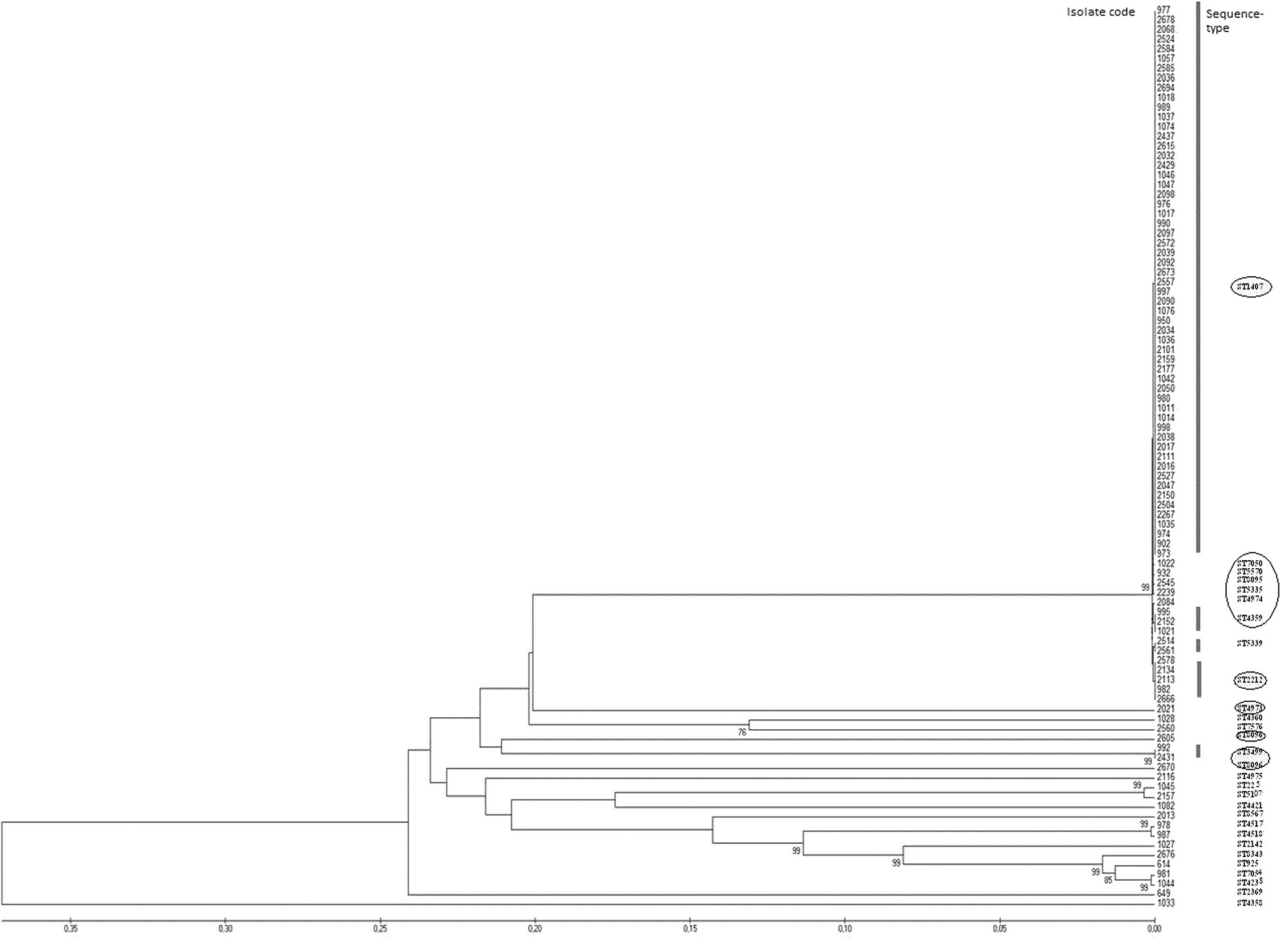
Phylogenetic tree obtained with Unweighted Pair Group Mean Average (UPGMA) clustering method using MEGA software [version 5.2; http://www.megasoftware.net] showing the relatedness of both *porB* and *tbpB* alleles (NG-MAST analysis) for 93 *N. gonorrhoeae* isolates. Circles indicate Sequence Types (STs) belonging to genogroup (G)1407

Figure 2 shows the similarity obtained from *tbpB* and *porB* sequences detected in all the isolates.

### MALDI-TOF MS analysis

MALDI-TOF MS allows to correctly identify, at the species level all the 93 isolates, of which 30.1 % with excellent score values (above 2.3); while one isolate was identified at genus level only (with a score of 1.987). The latter was resistant to cefixime and belonging to G1407. One minute per sample was the time estimated from the picking of the colony to obtain the identification results at the species level and 30 cents the cost per sample [21].

**Fig. 3:**
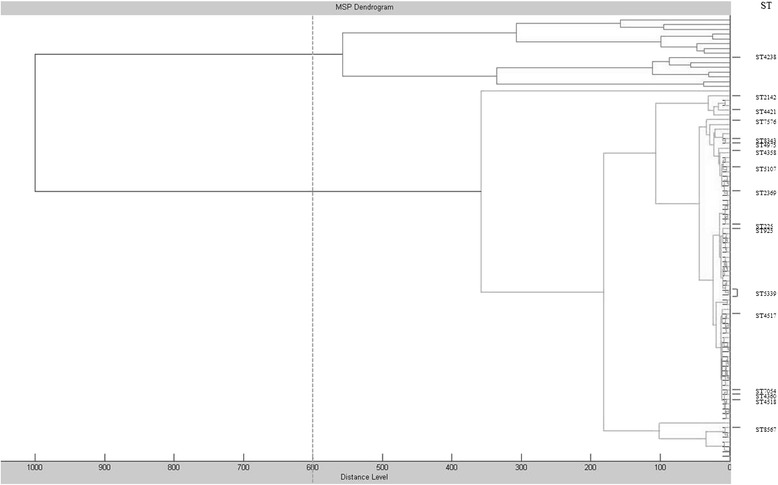
Cluster analysis of MALDI-TOF MS spectra obtained for 93 *N. gonorrhoeae* isolates. In the MSP dendrogram, relative distance between isolates is displayed as arbitrary units. Zero indicates complete similarity and 1000 indicates complete dissimilarity. The arbitrary distance level of 600 was chosen for isolates clustering evaluation. The STs listed in the Figure are different from the genogroup (G) 1407

As shown in Fig. 3, in relation to their mass signals and intensities, a hierarchic dendrogram clustered the 93 gonococci in two main groups: a large group (*n* = 77 isolates) it has been divided into two sub-clusters, the remaining 15 isolates were grouped into a smaller one. Conversely, 1 single isolate was grouped in a different cluster, according to the arbitrary distance level of 600 (Fig. 3). Dendrograms obtained from MALDI-TOF MS hierarchical cluster analysis and concatenation of *tbpB* and *porB* sequences were compared; the G1407 clustered as a single clone by NG-MAST, whilst turn to be divided in two groups by dendrogram obtained with MALDI-TOF MS analysis. In particular, in one cluster have been included isolates belonging to G1407 except for one that had the ST4232 with the *tbpB* allele 110 present among the G1407 isolates. In the second cluster, 60 out of 77 isolates belonged to G1407. The remaining 17 isolates defined small groups of 2 isolates, i. e. ST225 and ST5107 or ST4517 and ST4518 (Fig. 3).

## Discussion

Gonorrhea is considered the most common bacterial sexually transmitted infection and remains a public health concern due to the spread of antimicrobial resistance [3, 6, 33]. As typing tool, NG-MAST, is routinely used for the investigation of molecular epidemiology of gonococci as the reference method [14, 16, 34, 35]. Thus, it refers to the sequence variations in two hypervariable loci, *porB* and *tbpB,* coding for the gonococcal outer membrane porin and the β-subunit of the transferrin-binding protein, respectively [15, 29]. Furthermore, it is considered the current method of choice and widely used to identify the route of transmission [15]; several studies have shown a relationship between the NG-MAST and antimicrobial susceptibility profiles [10, 13, 14, 16, 34, 35].

Over the past few years, MALDI-TOF MS has become widely used for the rapid identification and typing of yeast and bacterial species in the clinical microbiology laboratory [23–25]. MALDI-TOF MS identification is based on the differences of whole cell proteins, which are mainly ribosomal proteins, by matching against a database.

In this study we evaluated the ability of MALDI-TOF MS for the rapid identification and cluster of gonococci particularly those belonging to G1407, the genogroup primarly associated with cefixime resistance. This clonal group represented the largest group in our study. Beyond this, gonococci were correctly identified by MALDI-TOF MS at the species level, except one, at the genus level only, with a score very close to the cut-off of 2.0. Compared to conventional biochemical methods used for the identification, mass spectrometry can be performed in few minutes. Further analysis would be required in order to evaluate the use of MALDI-TOF MS for diagnosis directly on clinical sample.

Moreover, the performance of MALDI-TOF MS for clustering of 93 *N. gonorrhoeae* isolates, already analysed by NG-MAST, were evaluated as a complement typing method, in particular for screening purposes.

The results showed that all the investigated gonococci were highly related and that those belonging to G1407 were divided into two distinct clusters by MALDI-TOF MS. Although, NG-MAST and MALDI-TOF mass spectrometry rely on quite different approaches, genome and proteome analysis respectively, the results suggest a role for MALDI-TOF MS for at least gonorrhoea identification. Nevertheless, as it is possible to argue, from the comparison between NG-MAST and MALDI-TOFdendrograms, not a reliable use for *N. gonorrhoeae* genotyping is attributable to the latter technique, even if inside the same genogroup, as identified by NG-MAST, the MALDI TOF MS clustered the isolates in more groups. In this context, we would like to mention the recent minireview by Spinali et al. [36]. In that article, it is well summarized the need to share guidelines for interpreting the data as already established for other techniques as Pulsed-field gel electrophoresis (PFGE). Furthermore, the potential of MALDI-TOF MS for typing purposes is contradictory from many examples, from *E. faecium* to *L. pneumophila*, [36].

Overall, some benefits may be attributed to the use of MALDI-TOF MS: the short time for the identification the low cost, the limited number of colonies needed in comparison with conventional methods.

## Conclusion

In summary the results are promising to consider MALDI-TOF MS for real-time identification of *N. gonorrhoeae* at the species level, with a reduction of time, consumables and skilled personnel compared to conventional methods. Finally, mass spectrometry approach needs to be further evaluated for gonorrhea typing purposes, even if a potential ability to discriminate among isolates belonging to the same genogroup was evinced.

### Availability of supporting data

The matrix obtained from the alignment of 93 *N. gonorrhoeae porB* and *tbpB* genes concatenation has been deposited in TreeBase link to the dataset DOI http://purl.org/phylo/treebase/phylows/study/TB2:S17893.

## Abbreviations

MDR: Multi-drug resistant
ECDC: European Centre for Disease Prevention and Control
AMR: Antimicrobial resistance
NAATs: Nucleic acid amplification tests
NG-MAST: *N. gonorrhoeae* multiantigen sequence typing
STs: Sequence types
G: Genogroup
MIC: Minimum Inhibitory Concentration
MALDI-TOF MS: Matrix-assisted laser desorption ionization-time of flight mass spectrometry
STI: Sexual Transmitted Infectious
DV: Dermatology-Venereology
EURO-GASP: European Gonococcal Antimicrobial Surveillance Programme
EUCAST: European Committee on Antimicrobial Susceptibility testing
WHO: The World Health Organization
UPGMA: Unweighted Pair Group Mean Average
HCCA: α-cyano-4-hydroxy cinnamic acid
MSP: Main Spectrum Profile
CCI: Composite correlation index analysis
ESCs: Extended-spectrum cephalosporins
PFGE: Pulsed-field gel electrophoresis
